# Impact of Traumatic Brain Injury on Neurogenesis

**DOI:** 10.3389/fnins.2018.01014

**Published:** 2019-01-09

**Authors:** Laura B. Ngwenya, Steve C. Danzer

**Affiliations:** ^1^Department of Neurosurgery, University of Cincinnati, Cincinnati, OH, United States; ^2^Department of Neurology and Rehabilitation Medicine, University of Cincinnati, Cincinnati, OH, United States; ^3^Neurotrauma Center, University of Cincinnati Gardner Neuroscience Institute, Cincinnati, OH, United States; ^4^Department of Anesthesia, Cincinnati Children’s Hospital Medical Center, Cincinnati, OH, United States; ^5^Department of Anesthesia, University of Cincinnati, Cincinnati, OH, United States; ^6^Center for Pediatric Neuroscience, Cincinnati Children’s Hospital Medical Center, Cincinnati, OH, United States; ^7^Department of Pediatrics, University of Cincinnati, Cincinnati, OH, United States

**Keywords:** epilepsy, traumatic brain injury, anesthetic neurotoxicity, spreading depolarization (SD), dentate gyrus, adult neurogeneses, granule cell

## Abstract

New neurons are generated in the hippocampal dentate gyrus from early development through adulthood. Progenitor cells and immature granule cells in the subgranular zone are responsive to changes in their environment; and indeed, a large body of research indicates that neuronal interactions and the dentate gyrus milieu regulates granule cell proliferation, maturation, and integration. Following traumatic brain injury (TBI), these interactions are dramatically altered. In addition to cell losses from injury and neurotransmitter dysfunction, patients often show electroencephalographic evidence of cortical spreading depolarizations and seizure activity after TBI. Furthermore, treatment for TBI often involves interventions that alter hippocampal function such as sedative medications, neuromodulating agents, and anti-epileptic drugs. Here, we review hippocampal changes after TBI and how they impact the coordinated process of granule cell adult neurogenesis. We also discuss clinical TBI treatments that have the potential to alter neurogenesis. A thorough understanding of the impact that TBI has on neurogenesis will ultimately be needed to begin to design novel therapeutics to promote recovery.

## Introduction

Adult neurogenesis in the hippocampal dentate gyrus is widespread in mammals. Generation of dentate granule cells occurs late in embryonic development, continues after birth, and persists into old age in most mammals examined (Amrein et al., 2011; Amrein, 2015; Ngwenya et al., 2015). Studies in rodents indicate that adult generated granule cells play a role in hippocampal dependent learning (Nakashiba et al., 2012; Danielson et al., 2016; Johnston et al., 2016). Whether neurogenesis continues into old age in humans remains controversial (Danzer, 2018a), with studies finding evidence for (Eriksson et al., 1998; Spalding et al., 2013; Boldrini et al., 2018) and against ongoing neurogenesis (Sorrells et al., 2018). Yet there is general agreement that dentate neurogenesis occurs in childhood and continues throughout young adulthood in humans, and that newly-generated neurons are poised to contribute to hippocampal function. At a minimum, therefore, traumatic brain injuries (TBIs) occurring during adolescence have the potential to disrupt this important process.

The generation, maturation, and integration of new neurons is critical for hippocampal function. This tightly regulated process, however, is easily disrupted by pathological events, such as TBI. In this review, we discuss the coordinated process of adult neurogenesis in the hippocampal subgranular zone (SGZ) and the impact that TBI and TBI treatments have on this process. An understanding of the regulation and dysregulation of neurogenesis is important for determining whether and how therapeutic interventions targeted at adult neurogenesis are useful for TBI treatment.

## Neurogenesis Is a Complex, Tightly-Regulated Process

**FIGURE 1:**
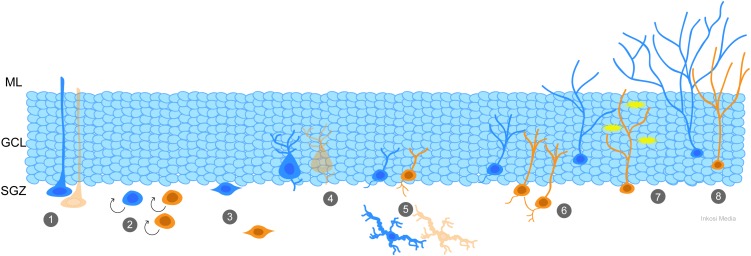
Generation and integration of adult-born granule cells is a coordinated process that is impacted by TBI. At each stage of adult neurogenesis, the normal process (blue) has potential to be altered by TBI (orange). (1) Quiescent radial neural stem cells (NSCs) in the subgranular zone (SGZ) can be depleted by frequent activation early in life, such as by TBI-induced seizures, leading to deficiencies with age. (2) TBI and its effects, including spreading depolarizations and seizures, cause an increase in proliferation of progenitor cells. (3) Newly-generated neurons migrate from the SGZ to the granule cell layer (GCL), and after TBI abnormal hilar migration is apparent. (4) Parvalbumin interneurons and (5) mossy hilar neurons are susceptible to cell death after TBI. Reduction in their numbers results in decreased GABAergic and glutamatergic (respectively) input to the newly-generated neurons. Newly-generated neurons show additional signs of aberrant neurogenesis such as abnormal connectivity (6), hyperexcitability (7) and inappropriate integration and dendritic maturity (8) which can be caused by changes in the environmental milieu.

Adult neurogenesis is characterized by multiple “control” points. The number of daughter cells produced by neural stem cells (NSC) located in the SGZ of the dentate gyrus can be modulated by the rate of cell proliferation and survival, while factors regulating fate specification control whether and how the new cells become neurons and integrate into the hippocampal circuitry (see recent review by Song et al., 2016). These control points can be regulated by signals released into the extracellular milieu by both neuronal and non-neuronal cells (Alenina and Klempin, 2015; Egeland et al., 2015), neurotrophic and transcription factors (Faigle and Song, 2013; Goncalves et al., 2016), neuroinflammatory mediators (Belarbi and Rosi, 2013), metabolic and hormonal changes (Cavallucci et al., 2016; Larson, 2018), and direct synaptic input from both glutamatergic and GABAergic neurons (Chancey et al., 2014; Alvarez et al., 2016; Song et al., 2016; Yeh et al., 2018). For additional information, the readers are referred to the excellent reviews cited for each mechanism, and the schematic in Figure 1. Critically, all of these factors can be disrupted by TBI, creating an environment in which immature granule cells and granule cell progenitors no longer receive the proper cues to guide their development.

## Neurogenesis Is Disrupted After Traumatic Brain Injury

Traumatic brain injury is particularly disruptive to the hippocampus due to its disparate pathomechanisms. Clinically, TBIs are classified as mild, moderate, or severe, however, the impact of TBI can include a variety of pathologies that are not sufficiently explained by clinical severity (Saatman et al., 2008). TBI can result from direct impacts or inertial forces. Pathologies include focal hemorrhage and contusions, diffuse pathology such as shear injury, and the myriad of pathoanatomic components seen in blast injury (Rosenfeld et al., 2013). Most human TBI involves a combination of forces and pathologies, and a variety of experimental TBI models exist to mimic these pathologies (Xiong et al., 2013). While not all TBIs directly involve the hippocampus, the structure nonetheless often exhibits signs of injury. For example, in the controlled cortical impact (CCI) model, which produces a focal cortical injury, cell death is apparent in the hippocampal dentate gyrus (Anderson et al., 2005). The contralateral hippocampus, remote from the injury site, can also show hippocampal injury and increased excitability after lateral fluid percussion injury (LFPI) (Tran et al., 2006). Involvement of the hippocampus raises the possibility that adult neurogenesis will be impacted.

Granule cell proliferation, survival, differentiation and maturation are impacted by TBI. Cells in the SGZ and inner granule cell layer undergo acute cell death after experimental CCI (Gao et al., 2008). In addition, however, TBI can also increase cell proliferation and neurogenesis (Dash et al., 2001; Chirumamilla et al., 2002; Urrea et al., 2007; Gao et al., 2009). Variable impacts on neurogenesis may reflect differences in injury severity (Wang et al., 2016). Notably, while there is speculation that increased neurogenesis may be beneficial (Rolfe and Sun, 2015), studies indicate that dentate gyrus neural progenitor cells are only capable of undergoing a finite number of replicative cycles before they terminally differentiate and become post-mitotic, ultimately depleting the regenerative pool (Encinas and Sierra, 2012; Neuberger et al., 2017).

Beneficial and pathological effects are also evident among the newly-integrated granule cells themselves. Inhibiting neurogenesis after CCI in mice (Blaiss et al., 2011) or after LFPI in rats (Sun et al., 2015) impairs spatial learning and cognitive recovery, suggesting that the new cells have positive effects. Consistent with this interpretation, treatment with growth differentiation factor 5 after CCI in mice was associated with increased neurogenesis and improved recovery (Wu et al., 2018). Similarly, optogenetic depolarization of immature granule cells and granule cell progenitors after LFPI in mice enhanced cell survival and maturation, while simultaneously improving cognitive measures (Zhao et al., 2018). However, while the axons of granule cells generated after LFPI follow the normal trajectory into the CA3 pyramidal cell layer (Emery et al., 2005; Sun et al., 2007), the cells can also exhibit morphological and physiological abnormalities. Following CCI, for example, newborn cells in mice exhibit abnormal dendritic branching (Villasana et al., 2015). Similarly, newborn granule cells in the LFPI model developed aberrant, hilar-projecting basal dendrites (Robinson et al., 2016). These newborn neurons also become ectopically localized to the dentate hilus (Robinson et al., 2016; Shapiro, 2017) or migrate too far into the granule cell layer (Ibrahim et al., 2016; Ngwenya et al., 2018). In line with the interpretation that neurogenesis can be pathological, treatment with a VEGFR2 antagonist after LFPI in rats suppressed injury-induced neurogenesis and prevented increases in seizure susceptibility (Neuberger et al., 2017), while treatment with the mTOR antagonist rapamycin after CCI in mice reduced neurogenesis, attenuated morphological abnormalities, and reduced seizure incidence (Butler et al., 2015). Hence, neurogenesis after TBI may produce a complex set of beneficial and pathological changes.

## Influence of Abnormal Electrical Activity After TBI

It has recently been shown that part of the pathophysiology after TBI is the occurrence of spreading depolarizations (SD). SDs are characterized by a massive wave of neuronal and glial depolarization that travels at 2–5 mm/min and is followed by electrical silence as neurons become temporarily refractory (Hartings et al., 2017). In patients with TBI, SDs are a predictor of mortality (Hartings et al., 2011) and are often the last electrical signal present in the brain just prior to death (Dreier et al., 2018). Their occurrence in migraine, however, suggests that the waves themselves can be relatively benign (Dreier et al., 2015). Studies have shown that this abnormal electrical activity causes an increase in neurogenesis (Urbach et al., 2008, 2016), the effects of which are currently unknown.

Acute seizures often occur immediately after TBI as a direct result of the traumatic force, and seizures are known to disrupt neurogenesis. Indeed, even a single, isolated seizure in a healthy animal is sufficient to increase granule cell neurogenesis (Bengzon et al., 1997). Seizures also disrupt granule cell integration, causing synaptic alterations (Jackson et al., 2012), abnormalities in dendritic structure (Murphy et al., 2012), migration defects and aberrant circuit formation (Scharfman et al., 2003; Parent et al., 2006; Jessberger et al., 2007b; Danzer, 2018b). In epileptic animals, seizure frequency is positively correlated with the frequency of abnormal, newborn granule cells (Hester and Danzer, 2013), suggesting that the number of seizures that occur following TBI is likely an important predictor of the degree of granule cell disruption.

## TBI Induced Changes to Dentate Gyrus Circuitry

In addition to seizure-induced cell death, direct effects of TBI and its immediate sequela can also cause death of key cellular components (Kharatishvili et al., 2006). Massive extracellular increases in glutamate follow TBI (McGuire et al., 2018), for example, and can cause excitotoxic injury. A wide variety of neurons are vulnerable. Dentate hilar neuron loss has been demonstrated after LFPI (Lowenstein et al., 1992; Grady et al., 2003) and includes parvalbumin positive, cholecystokinin positive, and GluR2/3 positive cells (Toth et al., 1997). Decreased parvalbumin immunoreactivity, for example, has been observed in the dentate following LFPI in rats (Huusko et al., 2015; Zhang et al., 2018) while time-dependent, interneuron-subtype specific changes have been described following diffuse TBI in rats (Carron et al., 2018). An observed reduction in spontaneous inhibitory post-synaptic current (sIPSC) frequency among mature granule cells months after LFPI suggests these changes have functional consequences (Pavlov et al., 2011), although impacts are temporally complex, as increases in sIPSC frequency have also been observed in granule cells after acute LFPI (Toth et al., 1997; Santhakumar et al., 2001; Gupta et al., 2012). Importantly, parvalbumin positive interneurons play key roles in regulating neurogenesis (Song et al., 2012, 2013) and their loss is likely to disrupt the process.

Glutamatergic mossy cells located in the dentate hilus are also extremely vulnerable to injury, including following TBI and seizures (Toth et al., 1997; Kienzler et al., 2009; Scharfman, 2016). Moreover, in the LFPI model, mossy cells that survive the insult are hyperexcitable (Santhakumar et al., 2000). Mossy cells directly excite granule cells, and are the first glutamatergic input to adult-generated granule cells (Chancey et al., 2014). The role of mossy cells is complex, however, as the neurons also indirectly inhibit granule cells by activating inhibitory interneurons which innervate granule cells (Scharfman, 2016). Both the direct glutamatergic and indirect GABAergic pathways have been shown to play a critical role in regulating granule cell neurogenesis (Yeh et al., 2018), so mossy cell loss and hyperexcitability following TBI will impact neurogenesis.

In addition to changes in local circuit neurons, TBI-induced changes in granule cell neurogenesis itself may exert effects on subsequent rounds of neurogenesis. Adult-generated granule cells transition through a distinct critical period during which they provide robust excitatory input to CA3 pyramidal cells, but only modest input to local circuit neurons mediating feedback inhibition (Temprana et al., 2015). As the cells mature, they integrate into and robustly activate inhibitory circuits within the dentate (Drew et al., 2016). The size of the newborn granule cell population at a distinct time point, therefore, may alter the development and integration of both more mature and less mature cohorts of granule cells. Taken together, therefore, newborn granule cell integration following TBI may reflect a complex interplay among disrupted circuits caused by interneuron loss, mossy cell loss and the size of previously-generated granule cell populations.

## The Effect of TBI Interventions

Clinical TBI interventions include a range of medically necessary and lifesaving measures, including surgery, anesthesia and treatment with neuroactive drugs to enhance care and recovery. Given the exquisite sensitivity of granule cell progenitors and immature granule cells to changes in the surrounding environment, these medications have the potential to exert both positive and negative effects on neurogenesis.

Anesthetic agents are a necessary part of clinical TBI treatment, yet they can have deleterious effects on NSCs and immature neurons. Studies in animal models demonstrate that clinically relevant doses of isoflurane induce neuronal apoptosis among newly-generated granule cells, with vulnerability peaking when the cells are about 2 weeks old (Hofacer et al., 2013; Jiang et al., 2016). This roughly corresponds to the period during which many newborn cells undergo natural apoptosis, suggesting that the anesthetic may artificially enhance the process (Deng et al., 2014; Lin et al., 2017).

Propofol, one of the most commonly used intravenous anesthetics in adult patients in both the operating room and the intensive care unit, has deleterious effects on adult neurogenesis. In the early postnatal period in rodents, propofol decreases the total number of granule cells and promotes dendritic spine loss (Huang J. et al., 2016). In adult animals, propofol impairs the maturation and differentiation of adult-born granule cells (Krzisch et al., 2013). After CCI, propofol attenuates the post-traumatic increase in adult neurogenesis and may contribute to cognitive impairment (Thal et al., 2014), although whether reduced neurogenesis and impaired cognition are mechanistically related in this model is not known.

Ketamine is a dissociative anesthetic whose impact on neuronal function is unresolved, yet has seen a recent resurgence in clinical use after TBI (Chang et al., 2013; Oddo et al., 2016). As an NMDA receptor antagonist, ketamine has been associated with both neurotoxic (Slikker et al., 2007; Yan and Jiang, 2014; Wang et al., 2017) and neuroprotective (Yan and Jiang, 2014; Bell, 2017) effects. The effect of ketamine on hippocampal neurogenesis is similarly mixed with evidence that ketamine interferes with proliferation of NSCs, but enhances neuronal differentiation (Huang H. et al., 2016; Soumier et al., 2016). The disparate effects appear dependent on timing and length of drug administration. After CCI, ketamine increased cell proliferation in the SGZ, decreased the number of newborn neurons, and ameliorated post-CCI cognitive deficits (Peters et al., 2018) suggesting that despite neurotoxic concerns, there may be beneficial effects. Indeed, ketamine is being evaluated as a promising therapy to halt SDs after TBI (Carlson et al., 2018; Hartings et al., 2018). As with propofol, the causal relationship between reduced neurogenesis and altered recovery has not been established. Moreover, the observation that propofol reduces neurogenesis and impairs cognition – while ketamine reduces neurogenesis and improves cognition – indicates that these associations should be interpreted cautiously.

Due to the occurrence of seizures following TBI, a variety of anti-epileptic drugs have been tried as potential therapies. Anti-epileptic drugs, however, often act by similar mechanisms as anesthetics, and can also induce apoptosis (Forcelli et al., 2011, 2012) and behavioral deficits (Gutherz et al., 2014) in young rodents. However, not all anti-epileptics have deleterious effects. Typical anti-epileptics phenobarbital and phenytoin have high side-effect profiles and are known to be pro-apoptotic (Bittigau et al., 2002), yet levetiracetam, a newer anti-epileptic medication that is being used with increased frequency in TBI patients (Jones et al., 2008; Szaflarski et al., 2010), may exert its effects by suppressing aberrant neurogenesis. For example, Sugaya et al. (2010) demonstrate in an animal model of status epilepticus that levetiracetam decreases the percentage of abnormally migrated hilar neurons. Levetiracetam has been shown to exert its effects on cell proliferation and neuronal differentiation by activation of the PI3/Akt pathway (Yan et al., 2018). Valproic acid, another commonly used anti-epileptic, also inhibits aberrant neurogenesis and induces neuronal differentiation. However, the mechanism of valproic acid may be through a PI3/Akt mediated epigenetic modification (Jessberger et al., 2007a; Zhang et al., 2017). This suggests that beyond suppression of seizures there may be a beneficial effect of certain anti-epileptic medications for patients with TBI.

Depression is a common post-TBI disturbance that is often treated with neuroactive medication. Post-TBI depression is generally managed with selective serotonin reuptake inhibitors (SSRIs), despite only minimal evidence of their efficacy in TBI (Yue et al., 2017; Kreitzer et al., 2018). It is suggested that there may be a causative relationship between depression and dysfunctional adult neurogenesis, with antidepressant medications exhibiting their effects via increases in neurogenesis (Santarelli et al., 2003; Eisch and Petrik, 2012; Yun et al., 2016). Chronic administration of the antidepressant medication fluoxetine increases NSC proliferation in the hippocampus (Malberg et al., 2000), however sertraline, another commonly used SSRI, appears to affect neuronal differentiation rather than proliferation (Peng et al., 2012). Antidepressant medications have also been shown to influence hippocampal neuronal plasticity by modulating dendritic spines (McAvoy et al., 2015), neurotrophic receptors (Rantamaki et al., 2007), and signaling cascades (Pilar-Cuellar et al., 2013) – all of which could impact neurogenesis. Finally, experimental TBI studies have shown increases in neurogenesis after administration of antidepressants, with varying effects on cognitive recovery (Han et al., 2011; Wang et al., 2011). These results suggest that the effects of antidepressants may extend beyond the treatment of depression. However, the timing of administration relative to the injury, the maturational state of adult-born granule cells potentially affected by the treatment, and whether the new cells are exerting net beneficial or pathological effects may all be important variables. Currently, there is insufficient data to determine when during the temporal sequence of events an intervention such as an SSRI might be most beneficial.

## Conclusion

Adult granule cell neurogenesis is exquisitely regulated by synaptic and extrasynaptic factors that can be directly impacted by TBI and TBI treatments. The process of neurogenesis includes proliferation, survival, maturation and functional integration. Just as each step of the process is regulated by ongoing activity in the neurogenic niche, changes in neurotransmission, electrical activity, and death of supporting cells can disrupt this process (Figure 1). The initial injury disrupts transmitter levels, and produces drastic changes in neuronal activity, including spreading depolarizations and seizures. The injury can also impair the function or induce the outright death of critical neuron populations providing input to new granule cells. Furthermore, exposure to anesthetic agents and other medically essential drugs alters the signals received by immature granule cells, and may have untoward effects on their survival or development. As it is becoming increasingly recognized that adult neurogenesis is an important component of TBI and cognitive recovery, disruption of this process has significant implications. Nonetheless, there does not appear to be a simple relationship between increased or decreased neurogenesis and improved or impaired recovery. Critical factors likely include the nature of the injury, the agent that alters neurogenesis, the timing of intervention, the sequence of the neurogenic process that is altered (e.g., proliferation vs. survival) and whether the new cells integrate into the hippocampal circuit in ways that are beneficial (improved cognition) or pathological (pro-epileptogenic). Despite these challenges, studies strongly suggest that neurogenesis is playing an important role in TBI, and therefore an understanding of how TBI and its interventions disrupt neurogenesis will be critical to guide the development of novel therapeutic approaches.

## Author Contributions

LN contributed to writing the manuscript and preparing the figures. SD contributed to writing the manuscript and preparing the figures.

## Conflict of Interest Statement

The authors declare that the research was conducted in the absence of any commercial or financial relationships that could be construed as a potential conflict of interest.
